# Continuous subcutaneous foscarbidopa/foslevodopa infusion as a therapeutic option in spinocerebellar ataxia type 8 presenting with early-onset parkinsonism

**DOI:** 10.1016/j.prdoa.2026.100496

**Published:** 2026-08-27

**Authors:** Francesco Onorati, Lorenzo Malfer, Lauren M. Jackson, Zhiyv Niu, Owen A. Ross, Rodolfo Savica

**Affiliations:** a Department of Neurology, Mayo Clinic, Rochester, MN, USA; b Department of Laboratory Medicine and Pathology, Mayo Clinic, Rochester, MN, USA; c Department of Clinical Genomics, Mayo Clinic, Rochester, MN, USA

**Keywords:** Early-onset parkinsonism, SCA8, Foscarbidopa/foslevodopa continuous subcutaneous infusion

## Abstract

In response to a case report on a patient with spinocerebellar ataxia type 8, presenting with early-onset parkinsonism and treated with deep brain stimulation, we report an analogous case, who responded optimally to continuous subcutaneous foscarbidopa/foslevodopa infusion.

Dear Editor,

We have read with great interest the article entitled “A case of spinocerebellar ataxia type 8 with clinical manifestations as early-onset Parkinson's disease” by Wang et al. [1]. The authors describe a remarkable case of SCA8 presenting with early-onset parkinsonism and propose deep brain stimulation (DBS) as an adjunctive therapy option. We report an analogous case in which the patient achieved an optimal response to continuous subcutaneous foscarbidopa/foslevodopa infusion.

The patient is a 27-year-old white man, who at first was referred to neurology evaluation in October 2020, with complaints of headaches, resting tremor, and stiffness. His past medical history was remarkable for attention deficit/hyperactive disorder (ADHD), whereas family history was non-contributory, with no first- or second-degree relatives manifesting neurological symptoms.

A DaT-scan performed in 2022 showed reduced radiotracer uptake in the right caudate nucleus and bilaterally in the putamen. In addition, metabolic testing of *catechol-O-methyltransferase (COMT)* revealed homozygosity for the reference allele rs4680 (c.472G/G; p.Val158Val), which is indicative of faster catecholamine catabolism. These findings, together with the patient's clinical presentation, led to the diagnosis of early-onset Parkinson's disease (EOPD), for which the patient was prescribed ropinirole at a total daily dose of 6 mg.

In January 2023, the patient was referred to our clinic for neurological evaluation. At examination, the patient demonstrated moderate left-sided bradykinesia, with mild rigidity and gait imbalances. Non-motor assessment revealed mild constipation and urinary incontinence. Depressed mood, anxiety, and apathy were also reported. MDS-UPDRS III and IV scores in the off-state were respectively 22 and 10, and Hoehn Yahr stage was 1.5.

Autonomic reflex screen showed no relevant abnormalities. Given the limitations of short-read sequencing in detecting repeat expansions, next generation long-read whole genome sequencing was performed, and identified a pathogenic *ATXN8/ATXN8OS* expansion, with 71 CTG repeats, consistent with SCA8.

Brain MRI with and without contrast showed no evidence of cerebellar atrophy or other abnormalities suggestive of ataxia. Ropinirole was discontinued due to increased risk of impulse control disorder, and a carbidopa/levodopa trial was initiated, starting from 150 mg/day and up titrating to a total daily dose of 600 mg. The patient had a good motor response, but began to develop dyskinesias and wearing-off, for which entacapone was added at a total daily dose of 600 mg. The severity and the daily burden of dyskinesias and fluctuations were significantly impactful on his quality of life, and multiple attempts to adjust the medication dose/frequency and formulation were undertaken but failed to help his symptoms.

In June 2025, due to progressive reduction of on-state time and increased dyskinesias, the patient began treatment with a foscarbidopa/foslevodopa subcutaneous infusion (adjusted base flow rate of 0.29 ml/h, high flow rate of 0.31 ml/h, and low flow rate of 0.28 ml/h). Most recent MDS-UPDRS III and IV testing in on-state yielded a score of 5 and 4 respectively, corresponding to a 17- and 6-point improvement from baseline (score = 22, 10).

This case adds to the growing evidence that SCA8 may present with parkinsonism, as previously described [1], [2]. Recent literature has further expanded the phenotypic spectrum of SCA8 to include progressive supranuclear palsy- and corticobasal syndrome-like presentations [3], [4].

The SCA8 CTG/CAG expansion has a relatively high population prevalence and variable expressivity, with many individuals showing reduced penetrance and diverse clinical presentations [5]. While SCA8 is inherited in an autosomal dominant fashion, it is common for the disease to present sporadically, due to its incomplete penetrance, and asymptomatic carriers harboring 70 to 1300 repeat expansions have been reported [5]. The role of *ATXN8*/*ATXN8OS* in neurodegenerative processes and the highly variable presentation of SCA8 remains incompletely characterized, and as this is a rare condition, the mechanisms underlying its pathogenesis are still poorly understood.

In our case, the mutation was not paternally inherited, as the father tested negative; however, it remains unclear whether the expansion occurred *de novo* or was maternally transmitted, as the patient's mother did not undergo genetic testing. Importantly, neither the parents nor any close relatives had any symptoms or signs of any neurological condition.

Interestingly, Wang et al. [1] noted that their patient showed a clear improvement with DBS in the medication off-state, whereas on-state demonstrated no additional benefit compared to fast-release levodopa, which the patient still needs, albeit at slightly reduced doses.

Notably, no clear consensus exists regarding oral levodopa responsiveness in SCA8 presenting with parkinsonism. Both our case and the one presented by Wang et al. [1] demonstrated a good initial response to oral levodopa but subsequently required advanced therapies to manage motor fluctuations. Conversely, Kim et al. [2] described a similar patient who demonstrated sustained benefit from long-term oral levodopa without motor fluctuations.

In our case, the patient experienced significant improvement with continuous subcutaneous infusion, which was initiated to address the fast wearing-off and dyskinesias that had occurred with oral levodopa. DBS was also discussed as an alternative to manage motor fluctuations, but due to patient's preference and reduced invasiveness, subcutaneous foscarbidopa/foslevodopa infusion was ultimately preferred.

Unlike oral therapy, which is characterized by significant fluctuations in plasma levodopa concentrations, continuous subcutaneous foscarbidopa/foslevodopa infusion has been demonstrated to provide a more stable levodopa exposure [6]. In fact, continuous subcutaneous administration is not subject to the characteristic unpredictable intestinal absorption of oral levodopa. This pharmacokinetic stability has been linked to significantly increased on-state time without dyskinesias and concomitant off-state time reduction [6].

Another contributor to the early motor fluctuations and wearing-off phenomenon observed in this patient was the homozygosity for the *COMT* reference allele (rs4680; Val/Val). This genotype produces a more thermostable and enzymatically active form of COMT compared to the Met/Met or heterozygous genotypes, resulting in accelerated dopamine catabolism and clinically significant early wearing-off [7]. Young age of onset and high levodopa dose may have also contributed to the development of dyskinesias, as both are risk factors for motor complications [8].

Some limitations should be acknowledged. Although this patient harbored a pathogenic SCA8 expansion, and multiple similar cases have been documented, other genetic or non-genetic causes of EOPD cannot be entirely excluded due to the variable expressivity and incomplete penetrance of the disease.

In conclusion, both DBS and foscarbidopa/foslevodopa infusion appear to be valid therapeutic options for the management of SCA8 patients presenting with parkinsonism. In particular, continuous subcutaneous infusion represents a recently FDA-approved non-surgical and well tolerated alternative to DBS. Our findings underscore that parkinsonism can occur in SCA8 carriers and may warrant targeted evaluation as part of SCA8 expansion carriers. With the increasing use of genetic testing, particularly within families, early neurological evaluation and recognition of these features in at-risk family members may open avenues for earlier and more effective clinical management.

## CRediT authorship contribution statement

**Francesco Onorati:** Writing – original draft. **Lorenzo Malfer:** Writing – review & editing, Conceptualization. **Lauren M. Jackson:** Writing – review & editing. **Zhiyv Niu:** Writing – review & editing, Formal analysis, Data curation. **Owen A. Ross:** Writing – review & editing. **Rodolfo Savica:** Writing – review & editing, Visualization, Funding acquisition, Formal analysis, Data curation, Conceptualization.

## Ethical approval

The Institutional Review Boards of the Mayo Clinic approved this study and written informed consent for the use of medical information was obtained from the participant.

## Funding

This research did not receive any specific grant from funding agencies in the public, commercial, or non-for-profit sectors.

## Declaration of competing interest

The authors declare that they have no known competing financial interests or personal relationships that could have appeared to influence the work reported in this paper.
